# Imaging flowers: a guide to current microscopy and tomography techniques to study flower development

**DOI:** 10.1093/jxb/eraa094

**Published:** 2020-05-08

**Authors:** Nathanaël Prunet, Keith Duncan

**Affiliations:** 1 University of California, Los Angeles, Los Angeles, CA, USA; 2 Donald Danforth Plant Science Center, St. Louis, MO, USA; 3 Trinity College Dublin, Ireland

**Keywords:** Bioimaging, confocal, flower development, light-sheet, microscopy, optical projection tomography, optical sectioning, super-resolution, tomography, two-photon, X-ray microscopy, X-ray tomography

## Abstract

Developmental biology relies heavily on our ability to generate three-dimensional images of live biological specimens through time, and to map gene expression and hormone response in these specimens as they undergo development. The last two decades have seen an explosion of new bioimaging technologies that have pushed the limits of spatial and temporal resolution and provided biologists with invaluable new tools. However, plant tissues are difficult to image, and no single technology fits all purposes; choosing between many bioimaging techniques is not trivial. Here, we review modern light microscopy and computed projection tomography methods, their capabilities and limitations, and we discuss their current and potential applications to the study of flower development and fertilization.

## Teaser

We review modern light microscopy and computed projection tomography methods, their capabilities and limitations, and their current and potential applications to the study of flower development and fertilization.

## Introduction

Angiosperms are one of the most successful groups on Earth. They have colonized six continents and thrive in a wide variety of environments and climates. This evolutionary success story is largely due to their reproductive structures: flowers. Most flowers are comprised of four types of organs: sepals, petals, stamens, and carpels; yet, flowers are extremely diverse in size, color, symmetry, scent, and number of organs, suggesting myriads of variations on a core developmental theme. Flowers also have a major agroeconomic importance: >80% of our food comes directly from plants, the vast majority of it fruits and seeds, which are parts and products of flowers. It is therefore critically important to understand the mechanisms underlying flower development and fertilization.

Richard Feynman, a theoretical physicist and Nobel laureate, famously said: ‘It is very easy to answer many of these fundamental biological questions; you just look at the thing!’ As biologists, we have daily reasons to scoff at that statement; however, ‘looking at the thing’ is undeniably a powerful way to try to understand biological phenomena. Developmental biology relies heavily on imaging to investigate how networks of genes and hormone signaling control organogenesis. This requires a precise four-dimensional (4D) knowledge of the model studied (i.e. the knowledge of the morphology of the model and its evolution through developmental time), and the ability to map gene activity and hormone response within these 4D structures. Early studies in flower development (e.g. Bowman et al., 1991, 1992; Jack *et al.*, 1992; Tröbner et al., 1992) used mutant approaches combining SEM to study the morphology of wild-type and mutant flowers with *in situ* hybridization and immunostaining to localize the corresponding gene products. These techniques have significant limitations, which complicates access to precise 4D information: they lack cellular resolution and require fixation and, in the case of gene expression analysis, sectioning the specimen.

The development of optical sectioning techniques, and particularly laser scanning confocal microscopy (Amos *et al.*, 1987; White *et al.*, 1987; Amos and White, 2003), combined with the design of a wide variety of fluorescent proteins (FPs) (van Roessel and Brand, 2002), has made it possible to map gene activity and hormone signaling in 3D, with cellular resolution, in live specimens. Confocal microscopes have become the work horse of developmental biology laboratories, and have been extensively used to study flowers, and led to many advances in the field (e.g. Mayer *et al.*, 1998; Heisler *et al.*, 2005; Urbanus *et al.*, 2009; Chandler *et al.*, 2011; Milani *et al.*, 2014; Sun *et al.*, 2014; Prunet *et al.*, 2017; Yamaguchi *et al.*, 2017; Xu *et al.*, 2018).

However, confocal microscopy is just one of many imaging techniques that can be used to study flowers. Biomedical imaging has seen an explosion of new techniques and provided us with a variety of tools for developmental biology. Here we review imaging tools available to flower developmental biologists, and cover both imaging modalities—from light microscopy to computed tomography—and reporters and sensors to detect gene expression and hormone gradients and response.

## 3D imaging with optical sectioning

Unlike single cells, flowers are thick specimens: a stage 2 Arabidopsis flower bud, for instance, is ~40 μm thick, and rapidly grows in size as it develops (stages as described in Smyth *et al.*, 1990). Yet, Arabidopsis flowers are small compared with those of many other species. While widefield epifluorescence microscopy works well for thin specimens, out-of-focus light strongly reduces the contrast and resolution when imaging 3D, thick specimens (Webb, 1999). One option to circumvent this problem is to fix and section the specimen, image the serial sections, and computationally generate a 3D reconstruction. This approach is not compatible with live imaging and therefore not ideal to study development. The development of optical sectioning microscopy techniques in the 1990s has revolutionized developmental biology.

**Fig. 1.**
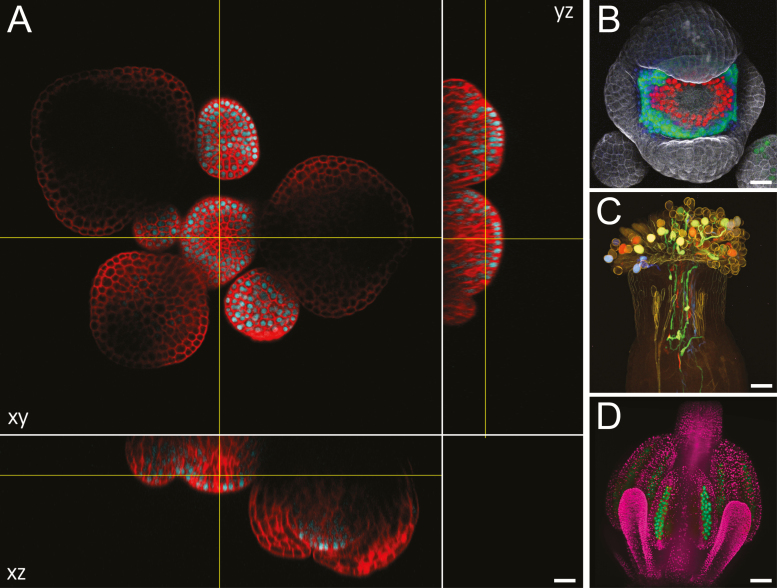
Arabidopsis flowers imaged with optical sectioning techniques. (A) Optical *xy* section and reconstructed *xz* and *yz* sections of a live Arabidopsis inflorescence expressing a transcriptional *SHOOT MERISTEMLESS* reporter (cyan) imaged with a point-scanning confocal microscope; cell walls were stained with propidium iodide (red); these images show the limitation of imaging depth with confocal microscopy. (B) Maximum intensity projection of a live, stage 5 Arabidopsis flower expressing a transcriptional reporter for *APETALA3* (*AP3*; green) and translational reporters for *AP3* (green) and *SUPERMAN* (red); cell walls were stained with propidium iodide (gray); note the differences in expression of the transcriptional and translational *AP3* reporters. (C) Maximum intensity projection of an Arabidopsis pistil pollinated with pollen expressing different transcriptional reporters (mTFP1, sGFP, Venus, and mApple) for *LAT52*, treated with ClearSee for 5 months, and imaged with two-photon excitation microscopy; this image, courtesy of Drs Yoko Mizuta and Daisuke Kurihara, was originally published in Kurihara *et al.* (2015). (D) Maximum intensity projection of a live Arabidopsis floral bud expressing reporters for the *ASY1* (green) and *H2B* (pink) genes; sepals were removed; image courtesy of Sona Valuchova and Pavlina Mikulkova. Scale bars=50 µm in (A–C), 100 µm in (D).

Confocal microscopy excites fluorophores within the specimen with a highly focused laser beam. Fluorescence emitted by the specimen is captured by the objective and filtered through a pinhole that only allows in-focus light to reach the detector (Webb, 1999; Murphy and Davidson, 2013). Successive optical sections are generated by scanning the laser beam over the specimen at different depths. These optical sections are then stacked together to generate a 3D reconstruction of the specimen, with a maximum optical resolution of ~250 nm laterally and ~500 nm axially (Table 1). Confocal microscopy has been extensively used to study flowers, and led to many advances in the field (e.g. Mayer *et al.*, 1998; Heisler *et al.*, 2005; Urbanus *et al.*, 2009; Chandler *et al.*, 2011; Milani *et al.*, 2014; Sun *et al.*, 2014; Prunet *et al.*, 2017; Yamaguchi *et al.*, 2017; Xu *et al.*, 2018) (Fig. 1A, B). However, this technique has some caveats: it is a point-scanning technique (i.e. images are created pixel by pixel, in contrast to widefield microscopy in which the image is acquired all at once by a camera), and is therefore intrinsically slow; while the out-of-focus light is not captured, for each pixel generated, a whole *z*-column in the specimen is excited by the laser, which causes the progressive photobleaching of successive optical sections, and can be toxic for live samples; this photobleaching and phototoxicity issue is reinforced by the need for high laser power to compensate for the light lost through the confocal pinhole.

**Table 1.** Characteristics of bioimaging techniques

Technique	Lateral resolution	Axial resolution	Imaging depth	Live imaging	Fluorescent reporters
Point-scanning confocal	250 nm	500 nm	100 µm	+++	Yes
Spinning disc confocal	250 nm	500 nm	100 µm	+++++	Yes
Two-photon	250 nm	500 nm	500 µm	++++	Yes, but not ideal for multiple colors
Light-sheet	300 nm	Depends on thickness of sheet	60 µm	++++++	Yes
SIM	100 nm	200 nm	15 µm	++	Yes
STED	20 nm	Variable but <500 nm	20 µm	+++	Yes
SMLM	20 nm	20 nm	5 µm	–	PALM, yes; dSTORM, no
OPT	1 µm	1 µm	15 mm	+++	Yes
Macro-OPT	6.5 µm	6.5 µm	45 mm	+++	Yes
XRM	500 nm	500 nm	1 cm^*a*^	–	No
XRT	20 µm	20 µm	1 m^*a*^	++	No

As far as possible the resolution and imaging depth values shown in this table are based on published data specific to flowers, or plant aerial tissues, and do not necessarily reflect the true resolution and imaging depth limit of the techniques. XRT and XRM resolution values for instance are estimates relating to imaging low-contrast biological samples; true instrument resolution for high-contrast materials, such as metals, ceramics, and geological samples, is higher.

^*a*^ Imaging depth is not relevant to X-ray imaging in the same way as with optical imaging methods. Rather, sample size varies inversely with achievable resolution and is dependent upon source–sample–detector geometry: the larger the sample or region of interest, the lower the voxel resolution.

Spinning disc microscopy is a faster, gentler approach to confocal microscopy. Instead of scanning the specimen with a laser beam, hundreds of discrete points are simultaneously excited through hundreds of pinholes spirally arranged on a rapidly rotating disc, and the light emitted by these points is collected through the same pinholes and captured by a fast camera (Oreopoulos *et al.*, 2014). As the disc rotates, the whole specimen is covered much faster than with point-scanning confocal microscopy to generate an optical section; moreover, cameras are more sensitive than point-scanning detectors, and less excitation light is needed. One disadvantage of spinning disc confocal microscopy compared with point-scanning confocal microscopy is the fixed sized of the pinholes, which cannot be adjusted to alter optical sectioning and resolution (Oreopoulos *et al.*, 2014).

Another way to generate optical sections of a thick specimen is to selectively excite in-focus parts of the specimen, which considerably reduces photobleaching and phototoxicity: each optical section is only excited once. This can be done with either two-photon excitation microscopy (also referred to as multiphoton excitation microscopy) or light-sheet microscopy (Huisken *et al.*, 2004; Helmchen and Denk, 2005; Murphy and Davidson, 2013; Weber *et al.*, 2014). Conventional fluorescence uses single-photon excitation: a fluorophore is excited with a photon of a specific wavelength, and rapidly emits a photon of longer wavelength and lower energy. In two-photon excitation, a fluorophore is excited by the simultaneous absorption of two photons of longer wavelength and lower energy than the photon it would absorb with single-photon excitation (Helmchen and Denk, 2005). Two-photon excitation is obtained by focusing a powerful femto-second pulse laser to a diffraction-limited spot within the specimen. Only in that spot are photons concentrated enough to generate two-photon excitation. Because the wavelength of the laser is too short to generate single-photon excitation of the fluorophore, excitation only occurs at this in-focus spot: there is no out-of-focus light. Two-photon excitation microscopy offers a similar resolution and speed to confocal microscopy (it is also a point-scanning technique) (Table 1).

Confocal and two-photon microscopy use the reflected light path (i.e. excitation and emission light are, respectively, shone onto the specimen through, and collected by, the objective lens). Conversely, light-sheet fluorescence microscopy separates the excitation and emission light paths: the specimen is illuminated by a sheet of light, generated either with a cylindrical lens, which focuses the light along one axis, or by rapidly scanning a laser beam along one axis within the exposure time of the camera (digitally scanned, or virtual light sheet); fluorescence emitted by the specimen is collected by an objective lens that is orthogonal to the illuminated plane and captured by a fast camera, generating an optical section (Huisken *et al.*, 2004; Weber *et al.*, 2014). 3D reconstructions can be obtained from stacking successive optical sections acquired from a single angle or through computational reconstruction of the specimen imaged at different angles—the latter option compensates uneven illumination of the focal plane as the light sheet is absorbed as it goes deeper into the tissues. Light-sheet microscopy offers an optimal lateral resolution of ~300 nm; axial resolution depends on the thickness of the light sheet, and can potentially be higher than that of confocal microscopy (Table 1). Light-sheet microscopy is much faster than point-scanning techniques. It is also gentler: cameras are much more sensitive than detectors used for point-scanning systems, and all the emitted light is collected (there is no pinhole), so less laser power is needed for illumination. These two characteristics make light-sheet microscopy an ideal optical sectioning technique for live specimens (Weber and Huisken, 2011; Weber *et al.*, 2014). It has emerged as a major tool for imaging live specimen in developmental biology in animals (Weber and Huisken, 2011), and has been very useful in plants to study roots, which are transparent (Ovečka et al., 2018). Applying light-sheet microscopy to aerial tissues, which are opaque and highly autofluorescent, has proven more difficult (Ovečka et al., 2015). However, light-sheet microscopy was recently successfully used to study germ cell development in Arabidopsis flowers over a period of days (Fig. 1D) (Valuchova *et al.*, 2020). We should expect a more widespread use of light-sheet microscopy to study flowers in the near future.

## 3D imaging with computed tomography

The word tomography comes from ancient Greek *tomos*, which means section. In a broad sense, any imaging technique that generates digital sections of a 3D object—including the optical sectioning techniques described above—could be considered tomography. Computed tomography, however, uses a reverse approach compared with optical sectioning: a 3D image of the specimen is computationally reconstructed from 2D projections that do not contain information about precisely where they come from within the specimen (Sharpe, 2004). The term tomography has typically been associated with X-ray tomography (XRT; also referred to as computed tomography, or CT scan), but many different tomography techniques use different part of the electromagnetic spectrum, including visible light in the case of optical projection tomography (OPT), but also sound waves or electric or magnetic fields (Sharpe, 2004). Here, we will focus on the use of XRT and OPT to study flowers.

XRT imaging has an X-ray source and detector enclosed in a lead cabinet to contain X-ray energy, and the patient or sample is placed between source and detector for imaging. The source generates X-rays that are directed through the sample toward the detector. Digital 2D images—radiographs—are projected onto the detector as X-rays pass through the sample and are differentially absorbed due to variation in sample density. Hundreds or thousands of radiographs are captured as the system or the sample rotates over (typically) 360°. All the 2D radiographs are then computationally reconstructed into a single 3D volume that can be manipulated and analyzed using advanced image analysis software. Human XRT imaging places the patient motionless in the center of the instrument while the source and detector rotate around the subject, whereas industrial XRT systems use a turntable upon which the sample rotates while positioned between source and detector.

The routine use of XRT in plant biology, particularly for imaging and analysis of complicated floral structures, is relatively recent. XRT imaging relies on differential density within a sample to generate an image. However, plant material typically is homogeneous low-density tissue, which makes high-resolution imaging difficult. Plants have been successfully imaged using large format industrial CT instruments simply by placing samples in appropriate devices that keep them stable during the course of the scan (see, for example, Bray and Topp, 2018; Roberston *et al.*, 2017; Li *et al.*, 2019). Nevertheless, there is a practical limit to sample size and resolution in large format instruments, at best approaching 20 µm voxel resolution for large plant samples (Table 1).

**Fig. 2.**
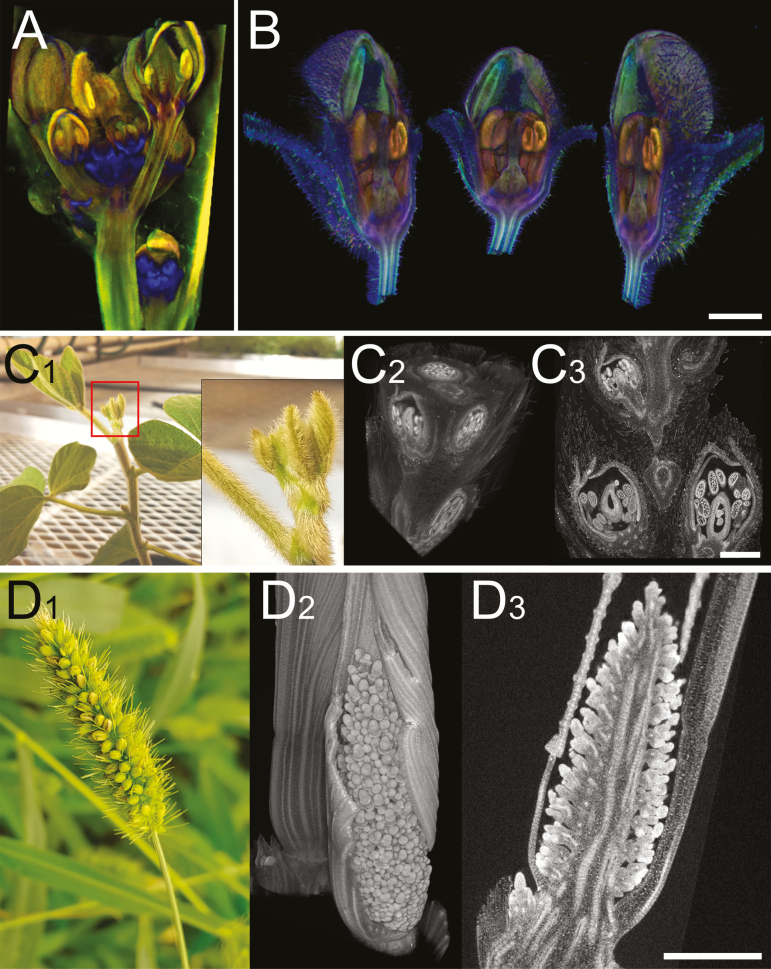
Computed tomography images of flowers. (A) Transmission OPT image of an Arabidopsis inflorescence expressing a GUS reporter for *LEAFY* (blue); image courtesy of Karen Lee. (B) Three views of an Antirrhinum flower imaged with emission OPT and virtual dissecting with a clipping plane to reveal internal structures; image courtesy of Karen Lee. C1–C3. Photograph (C1) and XRM images (C2 and C3) of a young soybean axillary bud containing numerous young florets that will eventually develop into soybean pods; C2 shows a virtual dissection with three clipping planes; C3 shows a computationally reconstructed section. (D1–D3) Photograph (D1), 3D computed reconstruction (D2), and computationally reconstructed section (D3) of young inflorescences from foxtail millet (*Setaria viridis*). Scale bars=500 µm.

For high-resolution imaging (i.e. microCT or nanoCT), floral structures are typically fixed and contrast enhanced (Staedler *et al.*, 2013). Recent examples have demonstrated the utility of fixation and contrast enhancement to image and analyze complicated floral and other plant biological tissues (Fig. 2C1–D3) (van der Niet *et al.*, 2010; Staedler *et al.*, 2013; Ijiri *et al.*, 2014; Wang *et al.*, 2015; Tracy *et al.*, 2017; Jeiter *et al.*, 2018; Mathers *et al.*, 2018; Staedler *et al.*, 2018; Duncan *et al.*, 2019). In particular, X-ray microscopy (XRM) has proven a valuable tool for imaging the complex biology of floral structures, providing high-resolution, data-rich biological information in 3D that is not practical or possible with other imaging technologies. The XRM adds a series of sophisticated microscope lenses to the traditional X-ray beam path (X-ray source–sample–detector); each lens has a coating that converts the X-ray signal into light, which is then magnified by the lens, and a high-resolution CCD camera functions as the detector to capture the final images. Again, hundreds or thousands of 2D digital radiographs are collected and computationally reconstructed into a detailed 3D volume with potentially submicrometer resolution for well-contrasted fixed samples. This provides excellent cellular detail in a full 3D volume with samples much larger and more complex than is typically possible with fluorescence microscopy. For example, entire large floral structures—1 cm^3^—can be imaged with XRM at 10 µm resolution, and specific regions of interest within that volume can be imaged at 1 µm resolution, all without physically cutting the sample into slices (Fig. 2C1–D3). This 3D imaging capability provides visualization and analysis of complicated floral morphology across scales, allowing unprecedented insight into floral developmental biology.

OPT uses light instead of X-rays. Like light microscopy, OPT can use either transmitted light (transmission OPT) or fluorescence (emission OPT). In transmission OPT, the specimen is placed between a widefield, diffuse light source and an optical lens system that focuses the transmitted light onto a CCD camera (Sharpe, 2004). In contrast, emission OPT uses UV light, and the emitted fluorescence is only collected from a certain angle, usually on the same side as for excitation. As in XRT, a 3D image of the specimen is computationally reconstructed from hundreds of projections acquired as the sample rotates over 360°, with a near-cellular maximum resolution (~1 µm voxel; Table 1) (Sharpe, 2004; Lee *et al.*, 2006). Lee *et al.* applied both transmission and emission OPT to plant tissues, including flowers (Fig. 2A, B), and developed a version designed to image larger specimens, called macro-OPT (Lee et al., 2006, 2017).

## From subcellular structures to large flowers—a matter of scale

While in most cases, imaging at cellular resolution is sufficient, some developmental studies require the ability to resolve much smaller, subcellular structures. However, the resolution of classic microscopy techniques is limited by the diffraction of light, which spreads the light from each point within the specimen into a diffraction pattern: the image of even an infinitely narrow point is thus not captured as a single point, but a point-spread function (PSF) (Murphy and Davidson, 2013). When two separate points of the specimen are too close, their PSFs overlap in the image, and the two points cannot be resolved as separate. The resolution limit depends both on the numerical aperture of the objective and the wavelength of light; typically, the resolution limit is ~300 nm for widefield microscopy techniques, and ~250 nm for point-scanning techniques.

Several microscopy methods, commonly referred to as super-resolution microscopy, push or bend the resolution limit. Structured illumination microscopy (SIM) uses the principle of Moiré fringes: the specimen is illuminated through a grid, which is rotated and translated over the specimen; the superimposition of the grid over the specimen causes the formation of coarse, resolvable details in the image: Moiré fringes. Because the spatial frequency of the grid is known, these resolvable patterns can be used to computationally deduce fine details in the specimen that are smaller than the resolution limit (Fiolka, 2014). SIM pushes the resolution limit down to 100 nm laterally and 200 nm axially (Table 1) (Komis *et al.*, 2015*b*). Other techniques such as stimulated emission depletion microscopy (STED) and single molecule localization microscopy (SMLM) completely circumvent diffraction to break the resolution limit. STED is a point-scanning technique that uses PSF engineering: the specimen is scanned with two laser beams; one excites the fluorophore, while the other, shaped into a donut surrounding the excitation beam, depletes fluorescence around the excitation point and restricts it to a very small spot (Murphy and Davidson, 2013). STED pushes the resolution limit down to 20 nm laterally (Table 1) (Komis *et al.*, 2015*b*). SMLM stochastically separates the emission of fluorophores in time, so that only a limited number of them emit light at any given time; in that way, the PSFs of neighboring fluorophores can be acquired separately instead of overlapping, and each fluorophore can be localized precisely to the center of its PSF (Murphy and Davidson, 2013). The final super-resolution image is generated from hundreds to thousands of successive images; SMLM is therefore a slow technique. Different SMLM modalities use different types of fluorophores. Photoactivation localization microscopy (PALM) relies on photoactivatable, photoswitchable, or photoreversible, genetically encoded FPs, and is therefore compatible with the imaging of live specimens. Conversely, direct stochastic optical reconstruction microscopy (dSTORM) uses immunostaining with organic fluorophores, which are induced to blink by high-level excitation in a redox buffer; dSTORM is thus not compatible with live imaging. SMLM pushes the resolution limit down to 20 nm (Table 1) (Murphy and Davidson, 2013).

Only a few studies have used super-resolution on plant tissues, and it has yet to be applied to flower development. However, super-resolution methods have the potential to resolve subcellular structures that are critical for flower development and their dynamics much better than traditional microscopy techniques. The cytoskeleton is one example: microtubules regulate plant development through their association with cellulose synthase and cell wall biosynthesis, as well as biomechanical constraints (Sampathkumar et al., 2013, 2014a, b; Hervieux *et al.*, 2016), and precisely resolving the organization of the microtubule network requires super-resolution (Komis et al., 2014, 2018). Another example is the flow of auxin through the PIN-FORMED (PIN) transporters: subcellular localization of the PIN proteins is highly polar, yet confocal microscopy does not have the power to resolve the plasma membranes and the shared cell wall of two neighboring cells; the localization of PIN proteins is usually inferred from the shape of the fluorescence from PIN fluorescent reporters, with no certitude regarding on which side of the cell wall the PIN proteins actually are. STED was used to precisely analyze the dynamic, polar distribution of PIN proteins in root tissues (Kleine-Vehn *et al.*, 2011), and similar approaches would shed a new light on auxin flows in developing flowers. SIM, STED, and PALM were also used for visualization of diverse subcellular compartments including the actin cytoskeleton, endoplasmic reticulum, endosomes, nuclei, plasma membrane subdomains, and nuclear nanodomains in living plant cells (Komis *et al.*, 2018).

On the opposite side of the scale, many flowers are too big to be imaged with a microscope. *Arabidopsis thaliana* has been by far the most studied plant model over the last 30 years. The small size of the Arabidopsis flower makes it possible to image it integrally within the field of view of a microscope objective, with cellular resolution, for a large portion of its development. This is not the case for bigger ornamental species such as *Antirrhinum majus* (snapdragon) and *Petunia hybrida*, which have long been used for flower development studies, or for crop species such as *Solanum lycopersicon* (tomato) or *Zea mays* (maize). Light microscopy is limited to relatively small specimens: the typical field of view of a 10× objective lens is ~2 mm. While it is possible to acquire and tile multiple, overlapping images to generate a 3D reconstruction, with cellular resolution, of samples that do not fit within this field of view, significantly larger specimens require the use of CT techniques.

OPT typically allows for the imaging of live specimens ranging from 0.5 mm to 16 mm in size, and therefore slightly too big for light microscopy, with near-cellular resolution (Fig. 2A, B), while macro-OPT can be used for specimens up to 60 mm, with a spatial resolution ranging from ~6.5 µm to 62.5 µm (Lee et al., 2006, 2017). XRM can be used to image specimens up to 1 cm^3^ in size with cellular resolution; a low-magnification scan (~20 µm voxel) of the entire sample can be combined with a high-resolution scan (500 nm voxel) (Table 1; Fig. 2C1–D3) to visualize features of interest. The primary drawback is the fixation and contrast enhancement required for XRM imaging; living tissue is difficult to immobilize sufficiently for the long scan lengths required for such high-resolution imaging. Finally, commercial XRT can accommodate much larger specimens, up to several meters in size, but without cellular resolution (20 µm voxel at best; Table 1).

## Imaging deeper in the tissues

Plants, and particularly aerial tissues, are difficult specimens in terms of light microscopy. Their epidermis is covered in a cuticle and, unlike animal cells, plant cells are surrounded by a cell wall of variable thickness, and contain a vacuole as well as plastids (plant cells range in size from ~3 µm to 100 µm, with cell walls ~0.1–10 µm thick). All these compartments and organelles have different refractive indices, making plant tissues strongly scattering (light rays are deviated at the interface between media of different refractive indices). Most floral organs also contain pigments that absorb light. Moreover, photosynthetic organs—which include sepals and carpels—are also strongly autofluorescent due to the presence of chlorophyll. This combination of scattering, absorption, and autofluorescence significantly hinders our ability to image flowers using optical microscopy: both quality and intensity of the signal degrade rapidly with imaging depth within plant tissues. Confocal microscopy typically allows for imaging at depths up to ~80 μm (Haseloff *et al.*, 1997), but imaging through sepals and carpels is further limited by chlorophyll absorption and autofluorescence (Fig. 1A). Similarly, light-sheet microscopy works well to image outer structures in flowers, but does not provide sufficient penetration to resolve inner tissues and organs through the sepals or carpels (Ovečka et al., 2015; Valuchova *et al.*, 2020).

One possible approach to circumvent this issue is to remove sepals or carpel valves to image the underlying tissues and organs. This can be achieved through manual dissection or laser ablation, and was successfully used in Arabidopsis to study the establishment of the boundary between stamens and carpels (Prunet *et al.*, 2017; Xu *et al.*, 2018), pollen tube growth, and male gamete release (Rotman *et al.*, 2003) with confocal microscopy, and germline differentiation with light-sheet microscopy (Fig. 1D) (Valuchova *et al.*, 2020), for instance. However, organ dissection is stressful for the specimen and results in less physiological imaging conditions. Another approach to get a better 3D reconstruction of deep tissues is to image the specimen from several angles, and combine the images computationally (Fernandez *et al.*, 2010; Ovečka et al., 2018; Valuchova *et al.*, 2020).

Two-photon microscopy is a better alternative to confocal and light-sheet microscopy for deep tissue imaging of intact, non-optically cleared specimens. It uses near-IR light, which penetrates deeper in tissues and scatters less than visible light, for excitation (Gilroy, 1997; Benninger and Piston, 2013). Moreover, two-photon excitation is restricted to the focal plane, thus each optical section is only excited once; this considerably reduces photobleaching compared with confocal microscopy, in which optical sections are excited repeatedly, causing the progressive bleaching of successive sections (Blancaflor and Gilroy, 2000). Two-photon microscopy allows for imaging several hundreds of micrometers deep within scattering specimens (Centonze and White, 1998; Helmchen and Denk, 2005). Two-photon microscopy enabled imaging in plant tissues at twice the depth obtained with confocal microscopy (Fig. 1C) (Mizuta *et al.*, 2015); it made it possible to image development processes that occur underneath several cell layers, such as pollen tube growth and double fertilization, *in vivo* (Cheung *et al.*, 2010; Mizuta *et al.*, 2015). Two-photon imaging at higher wavelength (>1000 nm) also strongly reduces autofluorescence in plant tissues (Mizuta *et al.*, 2015).

Even with two-photon imaging, scattering caused by the variety of refractive indices in plant cells limits deep-tissue imaging. Chemical treatments can be used to clear fixed tissues by reducing refractive mismatch and removing pigments. Chloral hydrate has long been used to clear plant tissues, but it is not compatible with the use of FPs (Kurihara *et al.*, 2015), which have become a major tool in developmental biology. Kurihara and colleagues used chemical screening to design ClearSee, a clearing solution for plant tissues that maintains the stability of FPs (Kurihara *et al.*, 2015). ClearSee has a high refractive index, and limits scattering; it is also highly efficient at removing chlorophyll, thus strongly reducing absorption and autofluorescence. ClearSee significantly increases confocal imaging depth in plant tissues, but the best results were obtained with two-photon microscopy of ClearSee-treated specimens (Fig. 1C): it is possible to image through an entire Arabidopsis pistil (~500 µm in diameter) (Kurihara *et al.*, 2015). To our knowledge, ClearSee has not yet been used in combination with light-sheet microscopy, but would undoubtedly increase the penetration depth of the light-sheet.

While it lacks cellular resolution, OPT allows for imaging much deeper than classic optical sectioning microscopy techniques (Sharpe, 2004). Regular OPT has been used to image plant specimens >10 mm thick, and macro-OPT plant specimens up to 45 mm thick (Lee et al., 2006, 2017). OPT and macro-OPT can be used to image live specimens; however, larger specimens typically need to be optically cleared to properly resolve deeper tissues (Lee et al., 2006, 2017). X-rays can penetrate much deeper into biological tissues than visible light, and XRT can therefore be used for intact 3D imaging for very thick specimens. XRM, however, is limited by the size of the specimen that can fit in the instrument, which can only accommodate samples up to 1 cm^3^ when sub-micron resolution is required. The most efficient system for X-ray tomography of plant biology across scales would combine a large format XRT—which can still image down to ~20–30 µm voxel (Table 1)—and the high resolution XRM.

## Adding the fourth dimension: live imaging

All the optical sectioning techniques described here, as well as OPT, are compatible with the imaging of live samples. However, they differ greatly in speed and photodamage of the specimen (Table 1). Point-scanning confocal and two-photon microscopy are slow, and confocal imaging can cause significant photodamage to the specimen, as successive sections are excited multiple times. Both methods can still be used for time-lapse imaging of flowers [for examples of time-lapse confocal studies of flower development, see Heisler *et al.*, 2005; Meyer *et al.*, 2017; Tsugawa *et al.*, 2017; detailed protocols for live confocal imaging of Arabidopsis (Fernadez et al., 2010; Barbier de Reuille *et al.*, 2015; Prunet *et al.*, 2016; Prunet, 2017) and Brachypodium flowers (O’Connor, 2018) are available, as are protocols for live confocal imaging of the shoot apical meristem of soybean and tomato (Geng and Zhou, 2019), which could easily be applied to imaging flowers]. However, samples can only be imaged every few hours at best; prolonged, repeated imaging of the specimen results in both phototoxicity and bleaching. Light-sheet fluorescence microscopy and, to a lesser extent, spinning disc microscopy, are much faster, making these methods better for live imaging. Indeed, light-sheet microscopy was used to image live Arabidopsis flowers nearly continuously over a period of 5 d without causing any significant bleaching, which would not be possible with point-scanning techniques (Valuchova *et al.*, 2020; protocols for live light-sheet imaging of plant tissues and flowers can be found in Ovečka et al., 2015, 2018; Valuchova *et al.*, 2020).

Computed tomography techniques require the acquisition of hundreds to thousands of projections to generate a 3D reconstruction of the sample, which makes data acquisition slow. Yet, OPT is compatible with time-lapse imaging (Table 1) (Lee et al., 2006, 2017). XRT can also be performed on live specimens. Various X-ray instrument manufacturers are exploring high-speed tomographic acquisition systems to allow some level of 4D XRT, but detector technology is still limiting this work to relatively low-resolution imaging (e.g. 100 µm voxel). XRM of plant samples, however, requires fixation and contrasting, and cannot be used for live imaging (Table 1) (Staedler *et al.*, 2013).

Super-resolution techniques are not all compatible with live imaging (Table 1). SMLM requires the acquisition of hundreds to thousands of images to reconstruct the final, super-resolution image and is therefore extremely slow and not geared towards live imaging (Murphy and Davidson, 2013). Still, PALM is technically compatible with the use of live specimen; dSTORM, however, uses fixed, immunostained samples. SIM also requires the acquisition of multiple images, but not nearly as many as SMLM (up to 25 images per channel per optical section depending on the number of phases used), and can be used for the live imaging of phenomena changing at moderate rates (e.g. microtubule growth) (Komis et al., 2015*a*, *b*). Finally, STED is a point-scanning technique and, as such, has the same restrictions as confocal microscopy; it is compatible with time-lapse imaging.

## Mapping gene activity and hormone signaling

The ability to transform plants (for a review of plant transformation methods, see Keshavareddy *et al.*, 2013) made it possible to generate transgenic reporter lines to analyze the patterns of gene expression. β-Glucuronidase (GUS) (Jefferson *et al.*, 1987), an enzyme that catalyzes the formation of a colored product from a colorless substrate, has been extensively used to study flower development (e.g. Jack *et al.*, 1994; Jenik and Irish, 2000; Ito *et al.*, 2003), but this approach suffers from the same limitations as *in situ* hybridization and immunostaining: it does not provide cellular resolution, and is not compatible with the study of live specimens.

The development of an extensive array of FPs of different colors, brightness, folding requirements, stability, and environment sensitivity from jellyfish *Aequora victoria*’s green fluorescent protein (GFP) and coral *Discosoma* sp.’s DsRed provided us with a wide variety of tools to design genetically encoded reporters that are compatible with biological imaging of live specimens (for the story behind the engineering of FP variants, see https://www.ibiology.org/talks/fluorescent-proteins/; for a guide of how to choose your FP, see Shaner *et al.*, 2005; for a database of FPs, see www.fpbase.org [Lambert, 2019]).

FPs have been used to generate reporters to monitor the expression of many genes that regulate various aspects of flower development, including the initiation of flower buds (e.g.Heisler *et al.*, 2005; Goldshmidt *et al.*, 2008; Besnard *et al.*, 2014), floral organ positioning (e.g. Chandler *et al.*, 2011), growth (e.g. Meyer *et al.*, 2017), identity (e.g. Urbanus *et al.*, 2009; Yamaguchi *et al.*, 2017), and polarity (e.g. Yamaguchi *et al.*, 2017), boundary formation (e.g. Takeda *et al.*, 2004; Prunet *et al.*, 2017; Xu *et al.*, 2018), and floral stem cell termination (e.g. Sun *et al.*, 2014; Yamaguchi *et al.*, 2017) using live confocal imaging. Transcriptional reporters (in which an FP gene is fused to the promoter of a gene of interest) and translational reporters (in which an FP gene is fused to both promoter and coding region of a gene of interest) can be used to map the domains where the corresponding mRNA and protein accumulate, respectively. Several genes involved in flower development show differences between these domains, due either to the ability of the protein to move between cells through plasmodesmata (e.g. *ARABIDOPSIS HISTIDINE PHOSPHOTRANSFER PROTEIN 6*) (Besnard *et al.*, 2014) or to protein instability in the absence of a partner (e.g. *APETALA3* and *PISTILLATA*; Fig. 1B) (Jack *et al.*, 1994; Krizek and Meyerowitz, 1996). Intercellular protein movement is also to be considered when designing transcriptional reporters, as the molecular size of single FPs allows them to move freely through plasmodesmata, generating a fluorescent domain that does not necessarily reflect the expression pattern of the gene of interest. This problem can be circumvented by adding a nuclear localization signal or an endoplasmic reticulum signal peptide to the FP, or using tandem FP fusions.

Phytohormones are also major regulators of flower development (Aloni *et al.*, 2006; Sundberg and ØOstergaard, 2009; Wybouw and De Rybel, 2019). Transgenic approaches have also been used to monitor hormone response and accumulation. Reporters for auxin (*DR5rev*) and cytokinin (*TCSn*) response were generated using synthetic promoters with multiple tandem repeats of response elements found in the promoters of genes responsive to hormones (Ulmasov *et al.*, 1997; Müller and Sheen, 2008; Zürcher et al., 2013), and fluorescent versions of these reporters are available (Benková et al., 2003; Brunoud *et al.*, 2012; Zürcher et al., 2013; Liao *et al.*, 2015). *DR5rev* and *TCSn* share common limitations: both promoters were designed from response elements identified in a single gene (Ulmasov *et al.*, 1997; Müller and Sheen, 2008; Zürcher et al., 2013), raising the question of whether these response elements are high- or low-affinity binding sites. While this question remains unsolved in the case of *TCSn*, it has been shown that *DR5* is a low-affinity binding site (Boer *et al.*, 2014). A higher affinity auxin response element has been identified (Boer *et al.*, 2014), and used to design a more sensitive reporter for auxin response: *DR5v2*, which reveals active auxin response in domains where auxin was predicted to accumulate but are not marked by the *DR5* reporter (Liao *et al.*, 2015).

Reporters based on hormone response elements are unlikely to reflect the complexity of hormone responses and do not directly reflect hormone accumulation patterns. Several hormone sensors have been designed to address this issue. An auxin sensor, *DII*, was built by fusing three Venus FPs to the auxin-interacting domain II (DII) of the IAA28 protein under the control of a constitutive promoter (Brunoud *et al.*, 2012). This domain is ubiquitylated in the presence of auxin and triggers the degradation of the protein. Absence of fluorescence in specific domains in plants expressing the *DII* reporter therefore indicates the presence of auxin (Brunoud *et al.*, 2012). An enhanced auxin sensor, *R2D2*, combines *DII-Venus* with *mDII-tdTomato*, in which a mutated, degradation-proof version of the DII domain is fused to a tdTomato FP (Liao *et al.*, 2015; for a comprehensive review on monitoring auxin concentration, transport, and response, see Parizkova *et al.*, 2017). The ratio between red and yellow fluorescence in different domains of plants expressing the *R2D2* reporter allows for the semi-quantitative measurement of auxin levels. Genetically encoded, ratiometric Förster resonance energy transfer (FRET) sensors can also be used to quantify the concentration of hormones (for a review on FRET sensors, see Okumoto *et al.*, 2012). FRET sensors combine two FPs, a donor and an acceptor, with a sensory domain. Binding of this sensory domain to its ligand induces a change of conformation that brings the donor and acceptor closer together, allowing the excitation of the acceptor FP by the light emitted by the donor FP. Changes in the ratio between donor and acceptor fluorescence intensity upon excitation of the donor reflect the concentration of the ligand. Such FRET sensors were recently designed for high-resolution quantification of abscisic acid, gibberellins, and now auxin (Jones *et al.*, 2014; Waadt *et al.*, 2014; Rizza *et al.*, 2017; Herud-Sikimic, 2020, Preprint). FRET sensors can also be used to monitor calcium concentration (Miyawaki *et al.*, 1997; Nagai *et al.*, 2004), and were used to detect rapid calcium fluctuations in ovules during double fertilization (Hamamura *et al.*, 2014).

Any light microscopy technique that produces optical sectioning can potentially be used to map gene expression and hormone signaling using multiple reporters. It is worth noting, however, that multicolor imaging is not trivial with two-photon microscopy, which uses a single, high-energy laser. To date, the majority of flower development studies have used confocal microscopy (e.g. Mayer *et al.*, 1998; Urbanus *et al.*, 2009; Chandler *et al.*, 2011; Sun *et al.*, 2014; Monniaux *et al.*, 2017; Prunet *et al.*, 2017; Yamaguchi *et al.*, 2017; Xu *et al.*, 2018) or two-photon microscopy (e.g. Mizuta *et al.*, 2015; Kimata *et al.*, 2016). While light-sheet microscopy was extensively used on underground tissues, it has been more difficult to apply to aerial tissues. The first study to use light-sheet microscopy to image flowers produced good surface rendering of the external morphology of the flower, but not of inner tissues (Ovečka et al., 2018); however, light-sheet microscopy was recently successfully used to study germ cell development in Arabidopsis flowers (Fig. 1C) (Valuchova *et al.*, 2020), and can be expected to be widely used in the future. OPT and macro-OPT can also be used to map gene expression and hormone response in flowers in 3D (Lee et al., 2006, 2017); it works both with reporters that catalyze the formation of a colored product from a colorless substrate, like GUS (with transmitted OPT; Fig. 2A), and with fluorescent reporters (with fluorescence OPT), and both types of reporters can be imaged in the same specimen. Conversely, X-ray-based imaging techniques do not currently allow for the imaging of either colored or fluorescent reporters. However, it may be possible in the future to design reporters based on enzymes catalyzing a reaction that produces contrasting agents and could therefore be visualized with CT scan and XRM. Further, the use of nanogold particles as contrast agents is being explored for contrasting plant and fungal structures (KD, personal communication) as has been done recently for studying soil properties with XRT (Scotson *et al.*, 2019). In the meanwhile, the recent development of correlative microscopy, and in particular fluorescence XRM (FXM), allows for combining fluorescence and XRM of the same specimen.

## Qualitative and quantitative image analysis

Modern bioimaging techniques rapidly generate large, complex 3D and 4D data sets, which are not trivial to analyze. A variety of open-access (e.g. MorphographX, FiJi, and MARS-ALT) and commercial (e.g. Aivia and Imaris) software makes it possible to segment cells, detect organelles, trace cytoskeleton filaments, and track cell lineages in developing specimens (Fernandez *et al.*, 2010; Barbier de Reuille et al., 2014, 2015). There is much more to microscopy and tomography images than meets the eye: providing that imaging is done correctly, image analysis software can also be used to extract a wealth of quantitative data from these bioimaging data, such as levels of gene expression, hormone accumulation and response, ratio between donor and acceptor fluorescence in FRET sensors, area and volume of different compartments, and cell growth (for examples of growth quantification in sepals and leaves, see Hervieux *et al.*, 2016; Kierzkowski *et al.*, 2019). Access to quantitative information over developmental time is critical to further our understanding of flower development.

## Concluding remarks

The explosion of new bioimaging technologies that we have seen over the last two decades is still ongoing: scientists are developing ‘mesoscopes’ that use larger objectives or mirrors instead of lenses to resolve cellular and subcellular details in specimens more than 1 cm in size (Perkel, 2019); expansion microscopy lets biologists resolve finer details by expanding their specimens (Wassie *et al.*, 2019); adaptative optics correct for light scattering in deep tissues (Marx, 2017). While some of these new techniques might prove difficult to adapt to plants (e.g. expansion microscopy), others will make their way into the plant field. In the meanwhile, this explosion of new imaging techniques has already provided biologists with invaluable tools to ‘look at the thing’. We now have the ability to study the development of live flowers, from a few dividing cells at the tip of a stem to mature flowers undergoing pollination and fertilization, with access to gene expression and hormone signaling, over a wide range of physical and temporal scales. When picking an imaging modality, we are also facing a complex choice. Ideally, we would like to generate sharp and well-contrasted 4D reconstructions of developing flowers at high spatial and temporal resolution, but no size fits all, and bioimaging requires compromises, between signal, speed, and resolution. We hope this review helps flower developmental biologists pick the imaging technique(s) that best fit their purpose.
